# Squid Pen Chitin Chitooligomers as Food Colorants Absorbers

**DOI:** 10.3390/md13010681

**Published:** 2015-01-20

**Authors:** Tzu-Wen Liang, Chih-Ting Huang, Nguyen Anh Dzung, San-Lang Wang

**Affiliations:** 1 Life Science Development Center, Tamkang University, No. 151, Yingchuan Rd., Tamsui, New Taipei City 25137, Taiwan; E-Mail: ltw27@ms55.hinet.net; 2 Department of Chemistry, Tamkang University, New Taipei City 25137, Taiwan; E-Mail: QQ1987pp@hotmail.com; 3 Institute of Biotechnology & Environment, Tay Nguyen University, Buon Ma Thuot 63000, Vietnam; E-Mail: nadzungtaynguyenuni@yahoo.com.vn

**Keywords:** chitosanase, squid pen, *Bacillus cereus*, chitooligomers, biosorbent

## Abstract

One of the most promising applications of chitosanase is the conversion of chitinous biowaste into bioactive chitooligomers (COS). TKU033 chitosanase was induced from squid pen powder (SPP)-containing *Bacillus cereus* TKU033 medium and purified by ammonium sulfate precipitation and column chromatography. The enzyme was relatively more thermostable in the presence of the substrate and had an activity of 93% at 50 °C in a pH 5 buffer solution for 60 min. Furthermore, the enzyme used for the COS preparation was also studied. The enzyme products revealed various mixtures of COS that with different degrees of polymerization (DP), ranging from three to nine. In the culture medium, the fermented SPP was recovered, and it displayed a better adsorption rate (up to 96%) for the disperse dyes than the water-soluble food colorants, Allura Red AC (R40) and Tartrazne (Y4). Fourier transform-infrared spectroscopic (FT-IR) analysis proved that the adsorption of the dyes onto fermented SPP was a physical adsorption. Results also showed that fermented SPP was a favorable adsorber and could be employed as low-cost alternative for dye removal in wastewater treatment.

## 1. Introduction

Chitosan is a partially deacetylated derivative of chitin, which consists of *N*-acetyl-d-glucosamine (GlcNAc) and d-glucosamine (GlcN) residues. Chitosanases are enzymes that catalyze the hydrolysis of the β-1,4 glycosidic bonds of chitosan. These enzymes have been found in abundance in a variety of bacteria [1,2,3,4,5,6,7,8,9]. Although chitosanases are efficient in producing chitosan [2,3,4,6], chitosan has been produced on an industrial scale mostly by the *N*-deacetylation of chitin using sodium hydroxide [10]. Among the natural chitinous resources, fishery wastes (crustaceans shells and squid pens) have an especially high content of chitin. Therefore, organisms that produce chitosanase with fishery wastes as the sole C/N source not only would solve an environmental problem but also could reduce the production costs of microbial chitosanase.

One of the most important and promising applications of chitosanases is the conversion of chitin-containing fishery waste into bioactive chitooligomers (COS). These oligomers have been shown to have potential to be the important pharmaceutical agents, these include antitumor, antioxidant [11], and antimicrobial activities [12,13,14,15]. Because the biological activities of COS are highly dependent on the degrees of polymerization (DP) and/or deacetylation (DD) [13], it is important to produce a well-defined COS mixture for specific uses, and study their structure-function relationship. One of the most important advantages of using chitosanase to produce COS rather than chemical operations (such as acid hydrolysis) is that it is a more environment-friendly process, and it generates a more defined COS mixtures. Therefore, obtaining an efficient mode for chitosanase production and the conversion of chitosan into bioactive COS will be highly beneficial for the chitin biotechnological industry.

In addition, chitin and chitosan have also been used in a wide range of applications, including effluent treatment for dye removal [16,17,18]. Most commercial systems currently use activated carbon as sorbent because of its excellent adsorption ability, but it is expensive and difficult to regenerate. In recent years, chitosan has been developed as a cheap and effective new alternative for dye removal. Chitosan is also a renewable resource and more environmental friendly than commercial materials. In a previous report, *L. paracasei* and *S. marcescens* have been used for the production of chitin from crab shell waste by successive fermentation [19]. Consequently, to decrease the cost of adsorbents for dye removal, the fermented fishery wastes in culture broth should also be able to be recovered for biological applications in dye removal. This idea inspired us to screen chitosanase-producing strains by using fishery wastes and to reclaim the wastes from the medium for dye removal.

**Figure 1:**
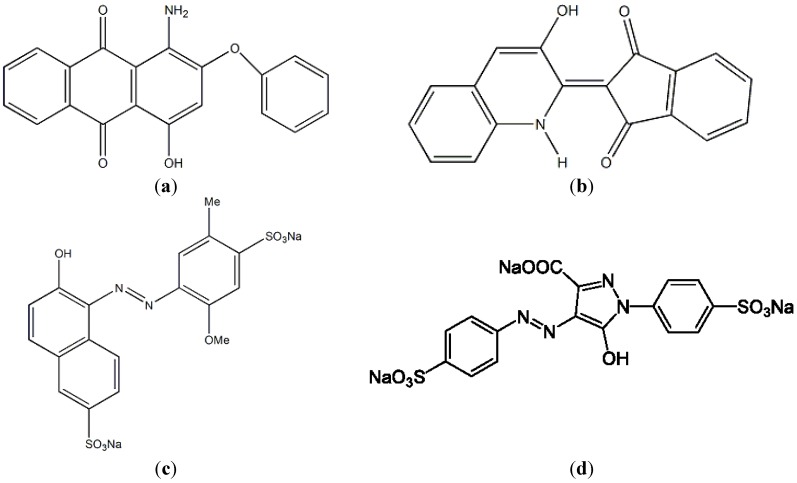
Chemical structures of disperse dyes and food colorants. (**a**) disperse red 60; (**b**) disperse yellow 54; (**c**) Allura Red AC; (**d**) Tartrazne.

A *Bacillus cereus* strain TKU033 that was capable of utilizing SPP to produce chitosanase was isolated from soil samples. The TKU033 chitosanase was purified, and its biochemical features were also characterized. In addition, the applications of the endo-type TKU033 chitosanase in functional chitooligomer production were also examined. To decrease the cost of adsorbents for dye removal, comparisons of the adsorption rates of the fermented SPP and the unfermented SPP for the disperse dyes (hydrophobic pigments, disperse red 60 (Figure 1a) and disperse yellow 54 (Figure 1b)) and water-soluble food colorants (Allura Red AC, R40 (Figure 1c); and Tartrazne, Y4 (Figure 1d)) were also undertaken.

## 2. Results and Discussion

### 2.1. Isolation and Identification of a Chitosanase-Producing Strain

The microorganisms were isolated from soil samples using the procedure described above. Among over 148 strains that were isolated in the laboratory and screened for chitosanase activity, the TKU033 strain was selected for further study. The TKU033 strain was maintained on nutrient agar and used throughout the study.

Strain TKU033 is a gram-positive and endospore-forming bacillus that contains catalase but not oxidase and is capable of growing in both aerobic and anaerobic environments. According to the results of the 16S rDNA partial nucleotide sequence (approximately 1.5 kbp) analysis, strain TKU033 was the most similar to *Bacillus* sp. According to the API identification, strain TKU033 was the closest to *B. cereus* with a 99.9% similarity. Therefore, the isolate was identified as *B. cereus.*

### 2.2. TKU033 Chitosanase Production and Purification

The production of chitosanase by strain TKU033 was investigated during 5 days of cultivation in the production medium. The 50 mL of basal medium (0.1% K_2_HPO_4_ and 0.05% MgSO_4_∙7H_2_O, pH 7) containing 1.5% SPP was the most suitable medium for the production of chitosanase by strain TKU033 at 37 °C. It was observed that the culture supernatant exerted strong chitosan degrading activities. Conclusively, the results suggested that the chitosanase from *B. cereus* TKU033 might be secreted extracellularly. Exponential growth of *B. cereus* TKU033 was observed for three days, and the stationary phase was reached after these three days. The highest chitosanase activity of *B. cereus* TKU033 was detected in the culture on the third day of bacterial growth (data not shown).

**Figure 2:**
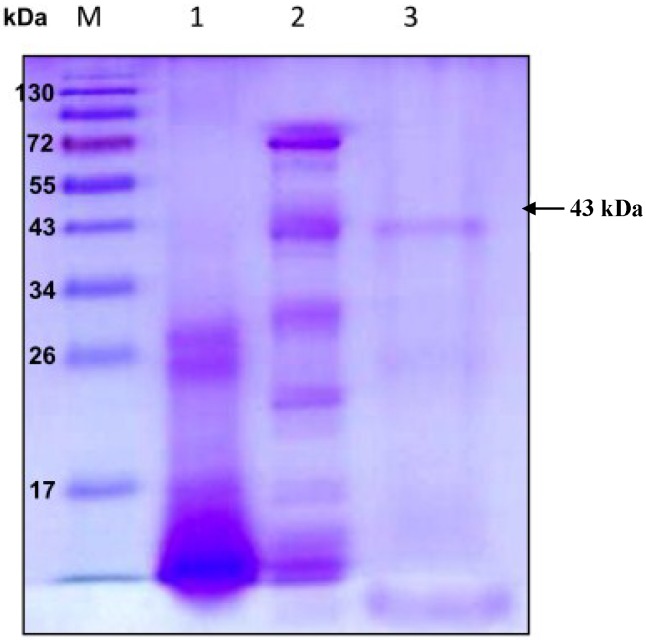
Sodium dodecyl sulfate polyacrylamide gel electrophoresis (SDS-PAGE) analysis of the chitosanase produced by *B. cereus* TKU033. Lanes: M, molecular markers (170, 130, 95, 72, 55, 43, 34, 26, 17, and 10 kDa); (1) crude enzyme; (2) adsorbed chitosanase fractions after DEAE-Sepharose CL-6B chromatography; (3) adsorbed chitosanase fractions after Macro-prep DEAE chromatography.

Extracellular chitosanase was purified from the culture supernatant of *B. cereus* TKU033 using a series of purification procedures. The TKU033 chitosanase was eluted in the DEAE-Sepharose CL-6B chromatography step with a linear gradient of 0–1 M NaCl in the same buffer. The eluted peak fractions were pooled for further purification. After the Macro-prep DEAE chromatography step (data not shown), approximately 5.8 mg of TKU033 chitosanase was obtained (Table 1). A summary of the purification process is presented in Table 1. The purification steps were combined to give approximately an overall 10.4-fold purification of the TKU033 chitosanase. The overall TKU033 chitosanase activity yield was 2% with a specific activity of 0.052 U/mg. The molecular mass of the TKU033 chitosanase was approximately 43 kDa as confirmed by the SDS-PAGE (Figure 2), which corresponded to the gel-filtration chromatography. The molecular mass of the TKU033 chitosanase (43 kDa) was similar to most chitosanases, which have a medium apparent molecular mass within the range of 20–75 kDa [7,20].

**Table 1:** Purification of the chitosanase from *B. cereus* TKU033 ^a^.

Step	Total Protein (mg)	Total Activity (U)	Specific Activity (U/mg)	Purification Fold	Yield (%)
Culture supernatant	4032.0	19.4	0.005	1.0	100
(NH_4_)_2_SO_4_ ppt	838.4	5.9	0.007	1.4	30
DEAE-Sepharose	46.5	1.7	0.037	7.4	9
Macro-prep DEAE	5.8	0.3	0.052	10.4	2

^a.^ *B. cereus* TKU033 was grown in 50 mL of liquid medium in an Erlenmeyer flask (250 mL) containing 1.5% SPP, 0.1% K_2_HPO_4_, and 0.05% MgSO_4_·7H_2_O in a shaking incubator for 3 days at 37 °C.

### 2.3. Effects of pH and Temperature

**Figure 3:**
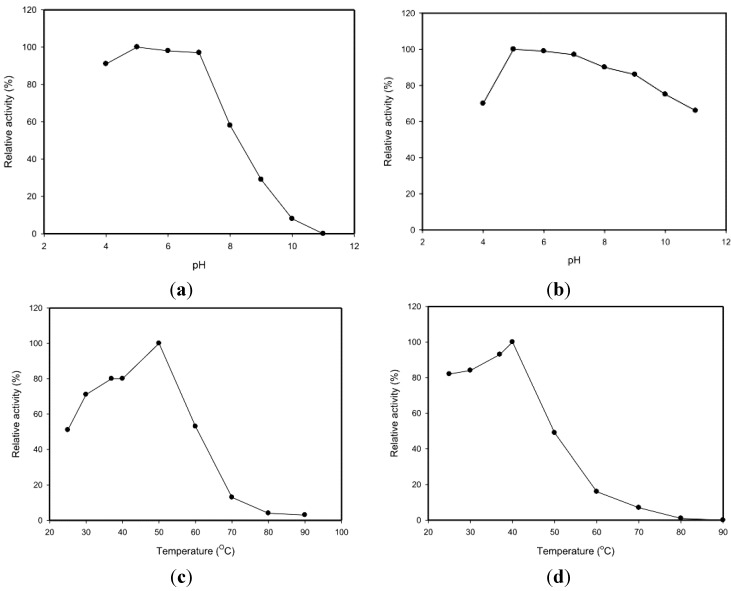
Effects of pH and temperature on the activity and stability of the chitosanase from *B. cereus* TKU033. (**a**) optimum pH; (**b**) pH stability; (**c**) optimum temperature; (**d**) thermal stability.

The pH activity profiles of the TKU033 chitosanase revealed maximum activity at pH 5 (Figure 3a). The optimum pH (pH 5) for TKU033 chitosanase activity was similar to that of most bacterial chitosanases, which display optimum activities at acidic pH values in a range from 4.5 to 6.5 [2,3]. The pH stability profiles of the TKU033 chitosanase were determined by measurement of the residual activity at pH 7 after incubation at various pH values at 37 °C for 60 min. The chitosanase was relatively stable at pH 5–9 and retained more than 85% of the initial activity in that range (Figure 3b). The TKU033 chitosanase became more sensitive to pH changes below pH 5 and above pH 9. The decrease of activity at lower and higher pH ranges may be due to the instability of the protein, rather than an acid-base catalytic mechanism, as reported in previous results [3,21].

The effect of temperature on the activity of the TKU033 chitosanase was also studied. The optimum temperature for the TKU033 chitosanase was 50 °C (Figure 3c). To examine the thermal stability of the TKU033 chitosanase, the enzyme solution in 50 mM phosphate buffer (pH 7) was allowed to incubate for 60 min at various temperatures; then, the residual activity was measured. Less than 10% of the residual activity could be detected after incubation at 70 °C under the conditions (Figure 3d). The TKU033 chitosanase was relatively stable from 25 to 40 °C and had 50% of its activity at 50 °C, but it was completely inactivated at 80 °C (Figure 3d). However, when the enzyme was incubated for 60 min at 50 °C and pH 5, in the presence of 0.3% (w/v) water-soluble chitosan, the residual activity of the enzyme was 93% (data not shown). These results indicated that the substrate could prevent thermal inactivation of the chitosanase activity. Enhancement of chitosanase thermostability by the substrate has previously been reported for chitosanase of the glycoside hydrolase (GH) family 8 from *Bacillus thuringienesis* [5] and the GH family 46 from *Bacillus subtilis* 168 [6].

### 2.4. Substrate Specificity

For the substrate specificity of the TKU033 chitosanase, chitin and chitosan with DD ranging from 60% to 98% were used as the substrates. The TKU033 chitosanase showed a remarkable specificity toward chitosan with DD ranging from 80% to 98%, although some detectable activity was observed against other substrates (data not shown). The TKU033 chitosanase exhibited no activity on 60% DD chitosan, α-chitin, and colloidal chitin. It has been reported that chitosanase is produced by using various carbon sources, for instance chitosan [2,22,23], wheat bran [1], glucose [4], and shrimp shell [24]. Most chitosanases exhibit a wide range of substrate specificities [25]. In this study, TKU033 chitosanase degraded chitosan specifically and cleaved chitosan with maximal activity on polymers of DD ranging from 80% to 98%. The chitin and highly acetylated chitosans appear to be poor substrates because they contain insufficient glucosamine residues for optimal activity. Further research will be done to determine the structure of the resulting oligo-chitosan terminal units and the site of the enzyme cleavage. In previous reports, most bacterial chitosanases hydrolyze chitosan efficiently and chitin to a lesser extent [3]. These results indicate that the physical form of the substrate affects the rate of hydrolysis. Taken together, these results suggest that the TKU033 chitosanase produced in a specific manner from *B. cereus* TKU033 may be a useful tool for the chitosan biotechnological industry in the production of COS.

### 2.5. Effects of Various Inhibitors and Metal Ions

For the effects of metal ions on enzyme activity, the TKU033 chitosanase activity was measured in response to different metal ions and demonstrated that preincubating the enzyme with 5 mM Fe^2+^, Zn^2+^, Cu^2+^, or Mn^2+^ resulted in 30%, 26%, 82%, and 70% inhibition, respectively (data not shown). In previous reports, Cu^2+^ and Mn^2+^ could activate or inhibit different chitosanases [26,27,28,29,30,31]; however, both Cu^2+^ and Mn^2+^ inhibited the TKU033 chitosanase at 1–5 mM. The TKU033 chitosanase activity was markedly different from other chitosanases; therefore, we hypothesized that different metal ions might affect the TKU033 chitosanase activity by influencing the protein structure. The TKU033 chitosanase activity was also reduced after the addition of EDTA. However, the TKU033 chitosanase activity was not affected by phenylmethanesulfonyl fluoride (PMSF). Thus, PMSF could be used to control the crude extract serine protease activity and prevent exhaustive protein degradation during downstream processes. Furthermore, the effects of different surfactants (SDS, Tween 20, Tween 40, and Triton X-100) on the stability of the TKU033 chitosanase were also studied. A high stability was observed for the TKU033 chitosanase towards various surfactants (data not shown). Upon incubation with 0.5%–2% of Tween 40, the TKU033 chitosanase activity increased slightly with the concentration of the surfactant. These results may be based on the fact that surface-active reagents might increase the turnover number of chitosanases by increasing the contact frequency between the enzyme active site and the substrate, which is accomplished by lowering the surface tension of the aqueous solution. However, in the presence of 2 mM SDS, the TKU033 chitosanase activity retained 83% of its original activity level. These results suggest that the disulfide bond in the enzyme molecule is associated with its chitosanolytic activity.

### 2.6. COS Preparation and Product Analysis

**Figure 4:**
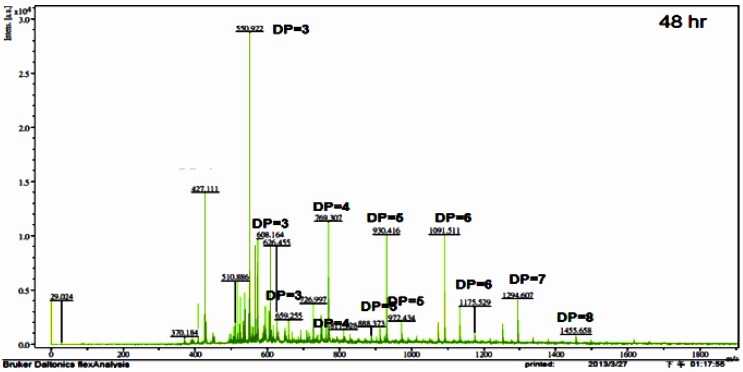
Matrix assisted laser desorption ionization time of flight mass spectrometry (MALDI-TOF-MS) of the chitooligomers (COS) mixtures obtained during the water-soluble chitosan hydrolysis for 48 h with the TKU033 crude enzyme. The proportion of low molecular weight oligomers was reduced by precipitation in the 90% methanol soluble/90% acetone insoluble fraction. The identified peaks are labeled with DP, in which DP indicates the degree of polymerisation.

To determine the applicability of the TKU033 chitosanase for the bioconversion of chitosan into oligosaccharides, the crude enzyme from *B. cereus* TKU033 was used in the experiments. The COS was produced from the water-soluble chitosan with the TKU033 chitosanase. The course of chitosan sample degradation was conveniently studied by measurement of the total and reducing sugars. The results revealed the total sugar and the reducing sugar of the sample as a function of reaction time. The chitosan sample total and reducing sugar values produced a similar pattern (data not shown). The total and reducing sugars increased, and the chitosan sample recovery decreased dramatically in the early reaction stage, which can be attributed to an endo-type degradation process. The TKU033 chitosanase was added into the reaction solution after 24 h and continued hydrolyzing until 48 h, but it did not improve the increase in total and reducing sugar levels (data not shown). Selective precipitation in 90% methanol and acetone solutions was performed to obtain low DP oligomers, as described earlier [13]. A product analysis of the 90% acetone solution precipitation by matrix assisted laser desorption ionization time of flight mass spectrometry (MALDI-TOF) revealed that various COS had DP values up to nine. The higher DP chitooligomers were precipitated as a light yellow powder in the methanol solution. The MALDI-TOF MS of the low DP oligomer fraction revealed pronounced differences among the crude enzyme-generated chitooligomers, as demonstrated for chitosan depolymerization in Figure 4. The hydrolysate ions present in the mass spectra were identified as sodium adducts [M + Na]^+^. Due to the signal interference of matrix (2,5-dihydroxybenzoic acid), DP < 2 oligomers could not be determined by this method. More information about the assigned structure of each signal at different hydrolysis times is given in Table 2. When the hydrolysis time was at 2 h, the hydrolysis products contained (GlcN)_4_; however, (GlcN)_4_ could not be observed during 4–48 h, indicating that the TKU033 chitosanase could cleave the GlcN-GlcN links of (GlcN)_4_ to (GlcN)_2_ or GlcN. The hydrolysates contained chitooligomers (GlcN-oligomers) and several partial *N*-acetylated forms (Table 2). The TKU033 chitosanase reaction product is a mixture of DP 3–9 hetero-chitooligomers (Table 2). These products were generated by hydrolysis of the water-soluble chitosan with the TKU033 chitosanase and are not a series of fully deacetylated GlcN oligomers. During hydrolysis, the *O*-glycosidic and the *N*-acetyl linkages between the residues can be hydrolyzed. These results indicate that the TKU031 chitosanase might hydrolyze chitosan in an endo-type fashion. From these results, chitosan hydrolysis by the TKU033 chitosanase, combined with a selective methanol precipitation, is a quick and simple method to obtain good chitooligosaccharide yields with DPs up to nine and low molecular weight oligomers.

**Table 2:** Ion composition of COS with DP below nine as assigned by the MALDI-TOF-MS spectra, prepared by enzymatic hydrolysis of the TKU033 crude enzyme for the indicated times and fractionated using selective isolation with 90% methanol and acetone.

*m*/*z*	Ion Composition	DP	Hydrolysis Time (h)					
			2	4	6	12	24	48
550	(GlcN)_2_-GlcNAc	3	+	+	+	+		+
608	GlcN-(GlcNAc)_2_	3		+		+	+	+
659	(GlcNAc)_3_	3	+			+		+
692	(GlcN)_4_	4	+					
727	(GlcN)_3_-GlcNAc	4		+	+			
769	(GlcN)_2_-(GlcNAc)_2_	4	+	+	+	+	+	+
811	GlcN-(GlcNAc)_3_	4		+	+	+		+
888	(GlcN)_4_-GlcNAc	5		+	+			+
930	(GlcN)_3_-(GlcNAc)_2_	5	+	+	+	+	+	+
972	(GlcN)_2_-(GlcNAc)_3_	5		+	+	+	+	+
1091	(GlcN)_4_-(GlcNAc)_2_	6		+			+	+
1133	(GlcN)_3_-(GlcNAc)_3_	6	+		+	+		
1175	(GlcN)_2_-(GlcNAc)_4_	6						+
1294	(GlcN)_4_-(GlcNAc)_3_	7	+	+	+	+	+	+
1455	(GlcN)_5_-(GlcNAc)_3_	8		+		+		+
1616	(GlcN)_6_-(GlcNAc)_3_	9		+		+		

### 2.7. Fermented SPP as a Biosorbent for Dye Removal

Chitin and chitosan, mostly prepared from shrimp shells, crab shells, and squid pens by chemical treatments, have been used in a wide range of applications, including effluent treatment for dye removal [16,17,18]. However, the costs of chitin-related preparations were far higher than those of their raw materials, marine chitin-containing byproducts. In this study, we chose SPP as the biosorbent for dye removal. For comparison, the three-day fermented SPP recovered from the culture broth of TKU033 was also analyzed. Appropriate amounts of the reclaimed SPP were added into 2 mL of dye-containing solutions. After shaking at 30 °C for 15 min, the reaction mixtures were centrifuged, and the residual dye in the supernatants was analyzed. The results showed that the color of SPP clearly changed from the colorless original to the color of the dye. The measured adsorption rates for all of the tested dyes also increased with the amounts of the fermented SPP added (data not shown). The maximal adsorption rate of the fermented SPP was 96% for the yellow disperse dye, followed by R40 (90%), the disperse red 60 (87%), and Y4 (68%) (Table 3). For both R40 and Y4 (water-soluble pigments), SPP (the unfermented SPP) showed a better adsorptive effect than that of the fermented SPP. As for the disperse dye (hydrophobic pigments), the fermented SPP exhibited a higher adsorption rate. Similar results were also found in our previous report [18] in which SPP showed a higher adsorption rate for water soluble colorants but a lower adsorption rate for the hydrophobic colorant (prodigiosin) than those of cicada casting and lactobacillus cells. The impact of SPP adsorption for water soluble colorants had been inferred to be related to the protein contained in SPP [18]. In this study, we compared the difference of the dye adsorption between the fermented SPP and the unfermented SPP, in addition to the difference of the protein in the two types of SPP; the difference of chitin structure in them should also be considered. Chitin is an amino polymer and has acetamide groups at the C-2 positions. The fermented SPP might result in a number of amine groups being introduced on the surface of the fermented SPP. The presence of these groups might be highly advantageous in providing distinctive adsorption functions for the disperse dyes.

**Figure 5:**
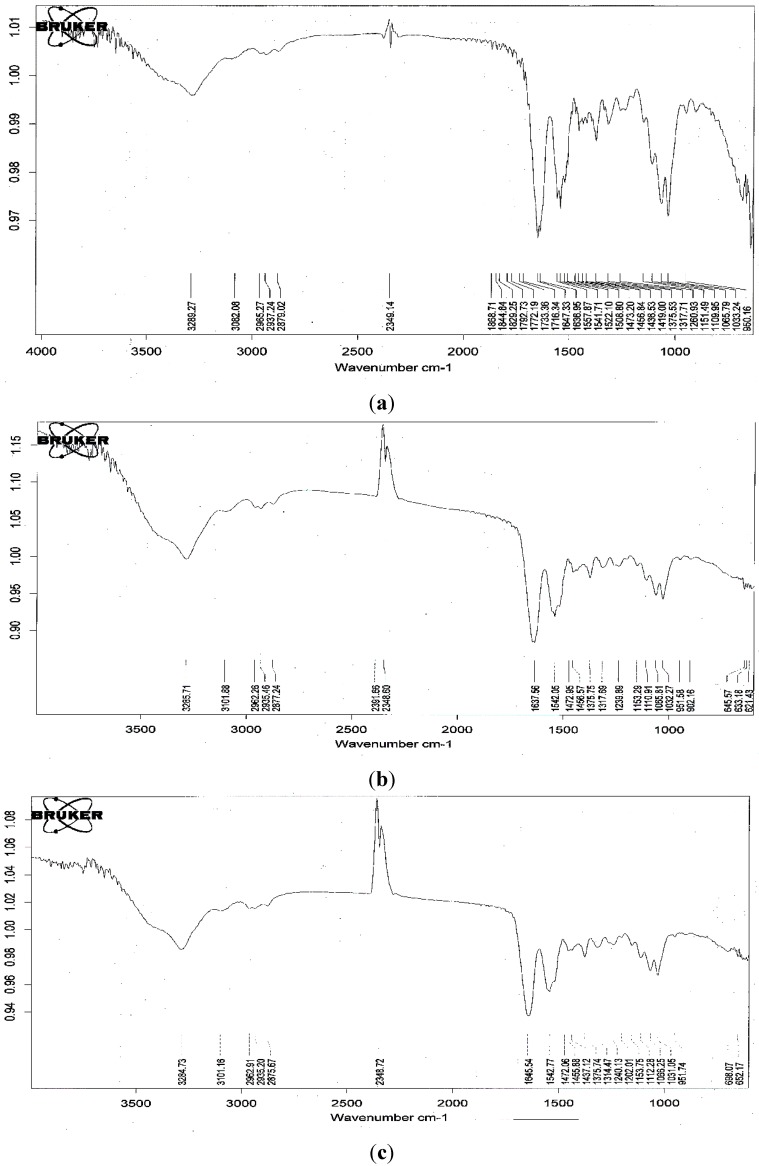
fourier transform infrared spectra (FT-IR) spectra of fermented SPP by *B. cereus* TKU033 before (**a**); and after the adsorption of R40 (**b**); and Y4 (**c**).

The fourier transform infrared spectra (FTIR) of the fermented SPP before and after the adsorption of a food colorant (R40 and Y4) are shown in Figure 5. There was no significant change in the spectra at wavenumbers 3289 cm^−1^ (-OH stretching vibration), 3082 cm^−1^ (C-H stretching), 2879 cm^−1^ (-CH_2_ stretching) and 1647 cm^−1^ (-NH stretching vibration) after the adsorption of the dyes. This implies that the adsorption processes represent a physical adsorption and may not involve a chemical interaction. These results provide a summary of the available information on SPP and its potential as a low-cost sorbent.

**Table 3:** Adsorption effect of squid pen powder (SPP) on the disperse dyes and food colorants.

Substrate	Adsorption (%)	
	Fermented SPP	Unfermented SPP
Disperse red 60	87	70
Disperse yellow 54	96	80
Food colorant (R40)	90	95
Food colorant (Y4)	68	82

## 3. Materials and Methods

### 3.1. Materials

The SPP used in these experiments was prepared as described previously [14]. The squid pens were purchased from Shin-Ma Frozen Food Co. (I-Lan, Taiwan). Diethylaminoethylcellulose (DEAE)-Sepharose CL-6B was purchased from GE healthcare UK Ltd. (Little Chalfont, Buckinghamshire, England). The weak base anion exchange Macro-prep DEAE was obtained from Bio-Rad (Hercules, CA, USA). Water-soluble chitosan with 90% DD was from Charming & Beauty Co., Taipei, Taiwan. Allura Red AC and Tartrazine were obtained from the First Cosmetics Works Company (Taipei, Taiwan). The disperse dyes were obtained from Widetex Biotech Co., Ltd. (Taoyuan, Taiwan). All other reagents used were of the highest grade available.

### 3.2. Screening and Identification of Chitosanase-Producing Strains

The microorganisms were isolated from soil samples collected at different locations in northern Taiwan. One gram of soil was ground in a porcelain mortar, 10 mL of sterile distilled water was then added, and the soil suspension was stirred. The SPP-supplemented media was inoculated with 0.5 mL of the soil suspension and incubated at 30 °C for 2 days. The single strain colonies that appeared were subcultured on agar plates containing 1% SPP, 0.1% K_2_HPO_4_, 0.05% MgSO_4_·7H_2_O, and 1.5% agar powder (pH 7). The plates were incubated at 30 °C for 2 days. The organisms obtained from this screening were subcultured in liquid media (containing 1% SPP, 0.1% K_2_HPO_4_, and 0.05% MgSO_4_·7H_2_O) in shaking flasks at 30 °C on a rotary shaker (150 rpm, Yih Der LM-570R). After incubation for 2 days, the culture broth was centrifuged (4 °C at 12,000 *g* for 20 min, Kubota 5922), and the supernatants were collected for the measurement of chitosanase activity using the procedure described below. The TKU033 strain showed the highest chitosanase activity, and it was isolated, maintained on SPP agar, and used throughout the study.

The bacterial strain TKU033 was identified on the basis of morphological, physiological, and biochemical parameters as well as on the basis of a 16S rDNA-based sequence analysis after PCR amplification with primers. The nucleotide bases of the DNA sequence obtained were compiled and compared with sequences in the GenBank databases using the BLAST program. Further identification of the strain TKU033 was performed using the analytical profile index (API).

The strain TKU033 grew on nutrient agar plates. The bacteria that grew on the surface of the agar plates were suspended by gentle mechanical agitation in 2 mL of sterile distilled water. This bacterial suspension was used to inoculate 50 CHB API strips (ATB system, bioMerieux SA, Marcy-I’Etoile, France) following the manufacturer’s instructions. The strips were incubated at 30 °C and observed after 16, 24, 40 and 48 h and compared to the API identification index and database.

### 3.3. Preparation of the TKU033 Chitosanase

For the production of the chitosanase, *B. cereus* TKU033 was grown in 50 mL of liquid medium in an Erlenmeyer flask (250 mL) containing 1.5% SPP, 0.1% K_2_HPO_4_, and 0.05% MgSO_4_∙7H_2_O (pH 7). One milliliter of the seed culture was transferred into 50 mL of the same medium and grown in an orbital shaking incubator for 3 days at 37 °C and pH 7.2 (the pH after being autoclaved was 7.5). After incubation, the culture broth was centrifuged (4 °C at 12,000 *g* for 20 min), and the supernatant was used for further purification via chromatography.

### 3.4. Measurement of Chitosanase Activity

The chitosanase enzyme activity was measured by incubating 0.2 mL of the enzyme solution with 1 mL of 0.3% (w/v) water-soluble chitosan (Kiotec Co., Hsinchu, Taiwan; with 60% deacetylation) in 50 mM phosphate buffer, pH 7, at 37 °C for 30 min. The reaction was stopped by heating the reaction mixture to 100 °C for 15 min. The amount of reducing sugar produced was measured with glucosamine as the reference compound [14]; one unit of enzyme activity was defined as the amount of enzyme that produced 1 μmol of reducing sugar per min.

### 3.5. Purification of the TKU033 Chitosanase

Ammonium sulfate (608 g/L) was added to the culture supernatant (450 mL). The resulting mixture was stored at 4 °C overnight, and the precipitate was collected by centrifugation at 4 °C for 20 min at 12,000 *g*. The precipitate was then dissolved in a small amount of 50 mM sodium phosphate buffer (pH 7) and dialyzed against the buffer. The resulting dialysate (the crude enzyme) was loaded onto a DEAE-Sepharose CL-6B column (5 cm × 30 cm) that had been equilibrated with 50 mM sodium phosphate buffer (pH 7). One peak exhibiting chitosanase activity was washed from the column with the same buffer, and the other chitosanase peak was eluted with a linear gradient of 0–1 M NaCl in the same buffer. The fractions of the two peaks containing chitosanase activity were independently pooled and concentrated using ammonium sulfate precipitation. The resultant precipitate was collected by centrifugation and dissolved in 5 mL of 50 mM sodium phosphate buffer (pH 7).

The obtained enzyme solution (the adsorbed chitosanase fractions from the DEAE-Sepharose CL-6B column) was then chromatographed on a Macro-prep DEAE column (12.6 mm × 40 mm) that had been equilibrated with 50 mM sodium phosphate buffer (pH 7). The chitosanase was eluted using a linear 0–1 M NaCl gradient in the same buffer. The fractions containing chitosanase activity were pooled and concentrated using ammonium sulfate precipitation. The pooled enzyme solution fractions were used as a purified preparation.

### 3.6. Protein Determination

The protein content was determined using the Bradford method with a Bio-Rad dye reagent concentrate and bovine serum albumin as the standard. Aromatic amino acids (phenylalanine, tyrosine, and tryptophan) have absorption band between 260 and 280 nm. To recover the protein after purification from the column chromatography, the protein concentration was estimated by comparing the absorbing band at 280 nm of the control tryptophan-containing standard [14].

### 3.7. Determination of Molecular Mass

The molecular mass of the purified chitosanase was determined using sodium dodecyl sulfate-polyacrylamide gel electrophoresis (SDS-PAGE) [14] with 12.5% acrylamide and 2.67% methylene bis acrylamide in a 0.375 M Tris-HCl buffer (pH 8.8) with 0.1% (w/v) SDS. Before electrophoresis, the proteins were exposed overnight to 10 mM phosphate buffer (pH 7) containing β-mercaptoethanol. The electrode buffer was composed of 25 mM Tris, 192 mM glycine and 0.1% (w/v) SDS (pH 8.3). The electrophoresis was performed at a constant current of 70 mA through the stacking gel and 110 mA through the resolving gel. After electrophoresis, the gels were stained with Coomassie Brilliant Blue R-250 in a methanol-acetic acid-water (5:1:5, v/v) solution and decolored in 7% acetic acid. The molecular mass of the purified chitosanase in its native form was determined using a gel filtration method. The sample and the standard proteins were applied to a Sephacryl S-100 column (2.5 cm × 100 cm) equilibrated with 50 mM phosphate buffer (pH 7). Bovine serum albumin (molecular mass: 67 kDa), *Bacillus* sp. α-amylase (50 kDa) and hen egg white lysozymes (14 kDa) were used as the molecular mass markers [14].

### 3.8. Effects of pH and Temperature on Enzyme Activities

The optimum pH values of TKU033 chitosanase were studied by assaying the samples at different pH values. The pH stability of TKU033 chitosanase was determined by measuring the residual activity at pH 7 as described above after the sample had been dialyzed against a 50 mM buffer solution of various pH values (pH 4–11) in seamless cellulose tubing (Daiichi Sankyo, Tokyo, Japan). The buffer systems used were acetate (50 mM, pH 4–5), phosphate (50 mM, pH 6–8) and Na_2_CO_3_-NaHCO_3_ (50 mM, pH 9–11). To determine the optimum temperatures for TKU033 chitosanase, the activity values of the samples were measured at various temperatures (25–90 °C). The thermal stability of TKU033 chitosanase was studied by incubating the samples at various temperatures for 60 min. The residual activity was measured as described above.

### 3.9. Effects of Various Chemicals and Surfactants on Enzyme Activities

The effects of metal ions (5 mM) were investigated using Mg^2+^, Cu^2+^, Fe^2+^, Ca^2+^, Zn^2+^, Mn^2+^, and Ba^2+^. The effects of enzyme inhibitors were studied using phenylmethylsulfonyl fluoride (PMSF) and ethylenediaminetetraacetic acid (EDTA). The effects of surfactants were also studied using SDS, Tween 20, Tween 40, and Triton X-100. The enzyme was pre-incubated with various chemicals and surfactants for 30 min at 25 °C, and the residual chitosanase activities were then tested.

### 3.10. Measurement of Total Sugars

To evaluate total sugars, the phenol-sulfuric acid method was used [32]. Briefly, 25 μL of 5% phenol was added to 1 mL of sample. After shaking, 2.5 mL of concentrated H_2_SO_4_ was added. The mixture was left to stand for 10 min and absorbance was read at 490 nm. Pure D-glucose was employed as standard.

### 3.11. Adsorption Effects of SPP and Fermented SPP for Dye Removal

Two food colorants and two disperse dyes were used as adsorbates. The dyes used in the experiments were Allura Red AC (R40) λ_max_ = 504 nm, Tartrazne (Y4) λ_max_ = 425 nm, Disperse Red λ_max_ = 530 nm and Disperse Yellow λ_max_ = 415 nm. Allura Red AC and Tartrazine were obtained from the First Cosmetics Works Company (Taipei, Taiwan). The disperse dyes were obtained from Widetex Biotech Co., Ltd. (Taoyuan, Taiwan). All these dyes were commercial grade and were used directly without further purification. The food colorants (R40 and Y4) and disperse dyes (red and yellow) (0.05%, w/v) were added to different concentrations of the fermented and unfermented SPP, respectively, and incubated at 25 °C on a rotary shaker (150 rpm) for one hour. After centrifugation, the residual pigments in the supernatant were then analyzed with a spectrophotometer.

## 4. Conclusions

In this study, we have succeeded in developing the efficient production procedure of chitosanase by *B. cereus* TKU033 using the cheap medium based on squid pen. This is different from most other reported chitosanase-producing strains which require chitosan as carbon/nitrogen source. The medium for TKU033 is obviously much simpler and cheaper. Besides, enzymatic hydrolysis by TKU033 crude enzyme could obtain the COS with DP 3-9. These results may be useful for biological applications in relation to enzyme and bioactive materials production. The fermented SPP in culture broth could also be recovered from the medium for biological applications in dye removal. It displayed a better adsorption rate for the disperse dyes than the water-soluble food colorants. The adsorptive capacities of fermented SPP on dye may have a potential application in the field of water treatment.
